# Thrombotic Events Develop in 1 Out of 5 Patients Receiving ECMO Support: An 11-Year Referral Centre Experience

**DOI:** 10.3390/jcm12031082

**Published:** 2023-01-30

**Authors:** Sasa Rajsic, Robert Breitkopf, Christopher Rugg, Zoran Bukumiric, Jakob Reitbauer, Benedikt Treml

**Affiliations:** 1 Department of Anesthesia and Intensive Care Medicine, Medical University Innsbruck, 6020 Innsbruck, Austria; 2 Institute of Medical Statistics and Informatics, Faculty of Medicine, University of Belgrade, 11000 Belgrade, Serbia

**Keywords:** ECMO, extracorporeal life support, thrombosis, thrombotic event, survival, adverse events, complications

## Abstract

Background: The use of extracorporeal membrane oxygenation (ECMO) for critically ill patients is growing rapidly given recent developments in technology. However, adverse events are frequently reported that have potentially devastating impacts on patient outcomes. The information on predictors and risk factors for thrombotic events, especially that focusing on the comparison of veno-arterial and veno-venous ECMO configurations, are still inconsistent and sparse; therefore, we aimed to close this gap. Methods: We performed a retrospective analysis of all patients on extracorporeal life support admitted to the intensive care units of a tertiary university center in Europe. Results: From 645 patients, 417 who received extracorporeal life support due to cardiogenic shock (290, 70%), respiratory failure (116, 28%) or hypothermia (11, 3%) were included. In total, 22% (92) of the patients experienced thrombotic events with a similar incidence in both ECMO configurations. Anticoagulation consisted of unfractionated heparin (296, 71%) and argatroban (70, 17%). Univariate Cox analyses identified hemoconcentration and increased maximal clot firmness (thromboelastometry) as risk factors for thrombosis. Moreover, the patients experiencing thrombosis had longer ECMO duration and intensive care stays. Conclusions: ECMO is a specialized life-support modality with a high risk of complications. A longer ECMO duration is associated with thrombosis occurrence in patients receiving ECMO support. Following hemorrhage, thromboembolic complications are common adverse events. However, in contrast to major bleeding, no impact on mortality was observed. The question arises if a protocol with less anticoagulation may have a role to play in the future.

## 1. Introduction

The use of extracorporeal membrane oxygenation (ECMO) for critically ill patients with severe respiratory or cardiac failure is growing rapidly given recent developments in technology. However, there is still debate regarding the benefits justifying its use [1,2,3,4,5,6,7,8,9,10,11].

Adverse events during ECMO support are frequently reported, which may affect patients’ outcomes significantly. Hemorrhage is one of the most frequent and serious adverse events during ECMO support followed by thrombotic events and sepsis [10,12,13,14]. The incidence of thrombosis is inconsistently reported, ranging from 10% up to 100% [12,15]. This discrepancy is most probably the consequence of its underestimation due to the lack of radiologic studies or post-mortem examinations in the majority of the predominantly retrospective studies [16]. Moreover, the lack of standardized definitions for thrombotic and bleeding events makes the comparison between studies even more complex.

Patients receiving ECMO support are at an increased risk of thrombotic events no matter the modality. While patients on veno-arterial ECMO (V-A ECMO) support are commonly subject to prior surgical intervention, patients on veno-venous ECMO (V-V ECMO) support often suffer severe infections. Due to the increased risk of bleeding, the former can be prone to overcautious anticoagulation, while the latter may suffer from thromboinflammation—a procoagulatory state triggered by hyperinflammation [17,18].

While multiple factors can account for adverse events, there are only a few that can be modified. In this regard, the mode and extent of anticoagulation might be the most accessible. Strict adaptation of systemic anticoagulation based on individualized monitoring presents the most adequate strategy according to the current recommendations [19,20,21]. Nevertheless, the evidence for the most suitable anticoagulation strategy and monitoring is still growing, and the results are controversial. Regardless of the ECMO modality (V-A ECMO or V-V ECMO configurations), recent studies have not shown any difference related to the risk for adverse events [20,21,22]. However, an increased risk for thromboembolic and hemorrhagic events seems more evident in hyper-coagulation states, such as in patients with COVID-19 receiving ECMO support, requiring significant anticoagulation treatment [13,23,24,25]. Therefore, sophisticated monitoring and management of the anticoagulation therapy may help improve patient outcomes by preventing and properly recognizing complications.

Comparing V-A ECMO with V-V ECMO configurations, this retrospective study aimed to evaluate the predictors and risk factors for thrombotic events during and shortly after ECMO support. Furthermore, we sought to analyze the clinical and demographic characteristics, the type and incidence of the adverse events, the anticoagulation regime, and the impact of thrombosis on the mortality in the patients receiving ECMO support.

## 2. Materials and Methods

### 2.1. Patient Selection

All patients requiring ECMO support between January 2010 and December 2020 at the Department of anesthesiology and intensive care medicine, Medical University Innsbruck, Austria were assessed for eligibility. The exclusion criteria included incomplete data sets, an age below 10 years, an ECMO duration of less than six hours and multiple ECMO initiations.

We prepared and revised our work according to the strengthening the reporting of observational studies in epidemiology (STROBE) statement—checklist of items (Supplementary Table S1).

### 2.2. Data Collection

The electronic medical records of the included patients requiring ECMO support were retrospectively reviewed. The obtained data included (1) sociodemographic data; (2) data regarding ECMO initiation and cardiopulmonary reanimation before or during ECMO implantation; (3) data regarding ICU treatment, such as ICU admission scores (Sequential Organ Failure Assessment—SOFA Score and the Simplified Acute Physiology Score III—SAPS III), anticoagulation, blood product transfusion, and detailed information on adverse events during treatment (thrombosis, bleeding, sepsis); (4) detailed coagulation status and other laboratory parameters; and, finally, (5) data on the cause and date of death from the clinical or post-mortem examinations. The laboratory parameters were recorded starting directly before the initiation of ECMO support and continued daily during the whole ECMO period.

This study was approved by the Ethics Committee of the Medical University of Innsbruck, Austria (1274/2019). The informed consent was waived due to the retrospective nature of the study.

### 2.3. Objectives and Outcomes

The primary objective was the identification of the predictors and risk factors for thrombotic events during and shortly after ECMO support. The secondary objectives included the comparison of clinical and demographic characteristics, including the type and incidence of adverse events. Finally, we analyzed the differences in the anticoagulation regime and the impact of thrombosis on mortality.

The outcomes comprised thrombotic events, hemorrhage, and sepsis. The data on thrombotic events (type, date of identification, and localization) occurring during the whole ECMO support period, including 14 days after ECMO termination (with observations of a maximum of 30 days after ECMO support initiation), were collected from the electronic medical charts and radiological reports. Thrombotic events were stratified according to their location into central (aorta, heart, and pulmonary artery) or peripheral arterial, venous or mixed thrombosis, multiple venous thrombosis and thrombus formation associated with central vascular catheters or ECMO cannula.

Hemorrhage was defined as major or minor according to the Extracorporeal Life Support Organization (ELSO) definition [19]. A major hemorrhage was defined as clinically perceptible bleeding (administration of two or more packed red blood cell concentrates or a hemoglobin decrease for at least 2 g/dL) within 24 h. Any noticeable hemorrhage that was not classified as a major event was defined as minor bleeding. The data on bleeding events were collected only for the period of the ECMO support duration, as any hemorrhage thereafter was not considered related to the ECMO. The information on sepsis was collected from the medical report and the list of diagnoses.

### 2.4. Anticoagulation during ECMO Support

Anticoagulation was performed according to the standard operating procedure protocol and was based on the ELSO Anticoagulation Guideline [19]. In general, ECMO circuits coated with unfractionated heparin (UFH) were used. Before ECMO cannulation, a UFH loading dose (50–100 IU/kg) was administrated to all patients if not already being on cardiopulmonary bypass. Continuous administration of UFH was usually chosen as the first-line anticoagulation (started with 5–20 IU/kg/h) with a goal aPTT of 50–70 s. In the case of a confirmed or a suspected heparin-induced thrombocytopenia type 2 (HIT 2) or insufficient response to heparin, argatroban was used (titrated to an argatroban blood concentration of 0.3–0.5 µg/mL). Anticoagulation was paused in cases of severe coagulopathy. We monitored anticoagulation every 30 min directly after its initiation until reaching a stable ACT, aPTT or argatroban blood concentration. Further on, routine controls were conducted every six to eight hours and consequently after any change of dosing. Point-of-care activated clotting time (ACT) was measured with every blood gas analysis, and aPTT was repeated if the ACT deteriorated.

Management of the patients undergoing elective cardiosurgical procedure and receiving antithrombotic therapy was performed respecting national and international recommendations [26,27]. All patients on a dual-platelet therapy and undergoing elective cardiosurgical surgeries continued using acetylsalicylic acid. The P2Y12 inhibitors were ceased prior to the intervention. In the case of emergency interventions, the effects of these medications were antagonized when possible. Within the prehospital treatment of patients with suspected acute coronary syndrome, the loading dose of acetylsalicylic acid and UFH (70 IU/kg) was administered. P2Y12 inhibitors were only used after consultation with the cardiologist on duty, either prehospital or directly before percutaneous coronary intervention.

### 2.5. Statistical Analyses

The statistical analyses were performed by a statistician using the R program (version 4.0.2, free software for statistical computing and graphics—R Core Team 2020: a language and environment for statistical computing; R Foundation for Statistical Computing, Vienna, Austria) and SPSS (Version 22.0. Released 2013, Armonk, NY, USA: IBM Corp.). A significance level of 0.05 was applied, and all statistical assessments were two-sided. Categorical data is presented as the frequency (percent) and continuous data as the mean with standard deviation or median with range (minimum–maximum) based on its distribution. Missing data were not analyzed. The independent sample t-test was used for parametric data and the Mann–Whitney U test for non-parametric data. The chi-square test and Fisher’s exact test were used to test group differences in the frequencies. The potential predictors of thrombotic events were analyzed in a univariate Cox proportional hazards model. Covariates with a significance level of *p* < 0.1 were included for analysis in a multivariate model. The Kaplan–Meier estimate was used to analyze the time to thrombotic events during the observation period.

## 3. Results

Over a period of eleven years, 645 patients required ECMO support due to refractory cardiogenic shock, severe respiratory failure or accidental hypothermia. In total, 417 patients met the inclusion criteria, and 92 (22%) experienced thrombotic events. The patient demographics and ECMO-related characteristics are presented in Table 1 and Table 2. The main indication for ICU admission was non-surgical related cardiac failure (n = 205, 49%) followed by respiratory failure (n = 108, 26%) and cardiac surgery (n = 90, 22%). The median duration of ICU stay was 18 (1–170) days.

ECMO support was predominantly initiated due to cardiogenic shock (n = 290, 70%) with a median duration of 6 (1–46) days; in total, 21% (n = 86) of the patients were mechanically resuscitated prior to ECMO initiation. A veno-arterial configuration occurred in 76% (n = 315) of cases, and a veno-venous configuration occurred in 25% (n = 102) of cases. Regarding anticoagulation, UFH was administered in 296 (71%) patients, and argatroban in 70 (17%) patients; in 50 (12%) patients, no anticoagulative drug therapy was given. Extracorporeal membrane oxygenation was discontinued due to successful weaning in 295 (71%) patients, fatality in 97 (23%) patients and bridge to another assist device in 18 (4%) patients. Of all patients, 148 (36%) died during the ICU stay (Table 3).

### 3.1. Thrombotic Events during ECMO Support

**Figure 1:**
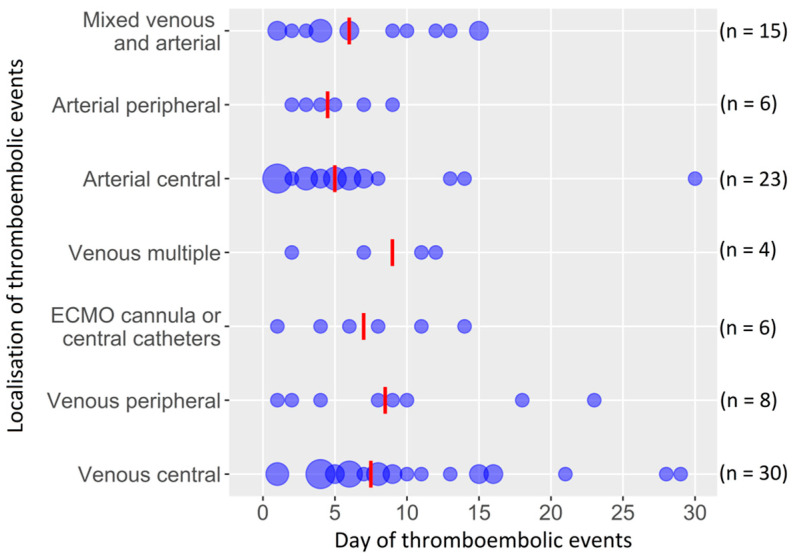
Locations of thrombotic events with the day of onset (circle size depends on the number of patients; red line presents the median; n = 92). Abbreviations: ECMO, extracorporeal membrane oxygenation. Arterial central (left ventricle, atrium, aortic valve and ascending aorta); venous central (right ventricle, atrium and pulmonary artery).

Thrombotic events occurred in 92 (22%) patients with a total of 58 (14%) patients in venous and 45 (11%) patients in arterial circulatory systems (Table 3 and Figure 1). The median time from ECMO initiation to detection of thrombosis was six days. Thrombosis was diagnosed in 53 (58%) patients during ECMO support and in 39 (42%) patients shortly after ECMO termination. An assessment of the laboratory parameters during ECMO support showed that the red blood cell count, hemoglobin, hematocrit, leukocytes and platelets were increased in the patients with thrombotic events (Supplementary Table S2). Besides thrombosis, the most frequent complication was hemorrhage (178, 43%).

Patients with thrombotic events received ECMO support for a significantly longer period (*p* = 0.001) with consequently longer ICU stays (Table 2). Interestingly, 35 of 92 patients with thrombosis experienced a concomitant hemorrhage, with major bleeding in 27 (29%) patients (Table 3).

The univariate Cox regression analyses identified chiefly laboratory variables to be associated with the occurrence of thrombotic events (i.e., increased hemoglobin, red blood cell count, leukocyte count or platelet count, InTEM and FibTEM maximal clot firmness; Table 4 and Supplementary Table S3).

Finally, a longer ECMO duration was associated with the occurrence of thrombosis (Table 5). The ICU length of stay, hemoglobin, hematocrit and InTEM Maximal Clot Firmness were excluded from the final model due to collinearity.

### 3.2. Thrombotic Events and Type of Anticoagulation

Argatroban was predominantly initiated in the patients with respiratory failure and UFH in the patients with cardiac non-surgical conditions. The patients receiving argatroban had higher SAPS III scores, longer ECMO support and longer ICU length of stay. Moreover, these patients suffered more often from sepsis (33% vs. 18%, *p* = 0.009, Supplementary Table S4).

Compared to the patients receiving anticoagulation, those without (due to severe coagulopathy) were older and predominantly on V-A ECMO support (n = 45; 90%) with 37 (74%) suffering cardiogenic shock. The ECMO duration was significantly shorter in these patients, and 24 (48%) patients died during ECMO therapy (Supplementary Table S4). Major hemorrhage occurred significantly more often (n = 21; 42%). Finally, not only ECMO mortality but also ICU mortality (n = 29; 58%) and one-year mortality (n = 33; 66%) were significantly increased in the patients without anticoagulation.

### 3.3. Thrombotic Events and ECMO Configuration

With regard to the ECMO modality (V-A ECMO vs. V-V ECMO), no significant difference in the incidence of thrombotic events was observed (Table 2 and Supplementary Figure S1). Compared to the patients with thrombotic events receiving V-A ECMO support, those receiving V-V ECMO support were younger (*p* < 0.001), had fewer surgical interventions (*p* = 0.001) and longer ICU stays (*p* = 0.034), as shown in Supplementary Table S5. Furthermore, the time from admission to ECMO initiation was significantly longer (mean: 0.1 vs. 2.2; *p* < 0.001), and the thrombotic event was detected significantly later (day 5 vs. 9; *p* = 0.003). The predominant location of the thrombotic event was the central venous system during V-V ECMO support (n = 14; 61%) but the central arterial system during V-A ECMO support (n = 20; 29%). The ICU and one-year mortality were comparable for the patients suffering thrombotic events, regardless of the ECMO modality (36% vs. 30%; *p* = 0.801 and 41% vs. 39%; *p* = 1.000).

## 4. Discussion

In this retrospective study from an ECMO referral center, 92 out of 417 patients experienced thrombotic events. The primary location was the central venous followed by the central arterial vascular systems or a combination of both. Anticoagulation was predominantly based on UFH (71%) followed by argatroban (17%). Compared to UFH, argatroban was more often administered in patients suffering from sepsis and respiratory failure, whereas no anticoagulant treatment (12%) was mostly chosen in patients receiving V-A ECMO support due to cardiogenic shock often also suffering major hemorrhage. The incidence of thrombotic events was comparable, regardless of the ECMO configuration. However, V-V ECMO support was more frequently associated with central venous thrombosis and V-A ECMO support with central arterial thrombosis. Neither thrombotic events in general nor thrombotic events with ECMO support, regardless of circuit configuration or anticoagulant agent, seemed to influence mortality.

### 4.1. Thrombotic Events during ECMO Support

Critically ill patients are at an increased risk for thrombotic events [28], even more during thromboinflammation, a well-established condition in patients with COVID-19 [17,18]. Moreover, the exposure of blood to artificial surfaces (e.g., ECMO) and surgical trauma further initiates and propagates the inflammatory cascade, additionally increasing the risk of thrombosis [29]. With regard to patients receiving ECMO support, the great intravascular surface area of the ECMO cannulas, partial low blood flow and stasis, indwelling central venous catheters together with immobility provide an ideal environment for thrombosis occurrence. Hence, patients receiving ECMO support are at a high risk of thrombotic events. Noteworthy, the patients requiring ECMO support analyzed by us were slightly overweight, which is in line with current literature [20,30]. As obesity is known to be an established risk factor for thrombosis [31], this may be seen as an underlying factor that cannot be modified.

The true incidence of thrombotic events during ECMO support still remains unknown. Recently, thrombosis incidence in V-V ECMO support was reported to range from 15% to 86% [12,13,20,23,32,33,34] and in V-A ECMO support from 16% to 46% [16,20,34,35], with our data located in the lower region. To date, only a few studies have investigated the risk factors for thrombosis, and a paucity of evidence regarding incidence, mortality risk and predictors remains. Furthermore, due to the lack of certain clinical manifestations, thrombotic events may be highly underestimated. In post-mortem examinations of 78 post-cardiotomy patients receiving ECMO support, only one-third of the nearly 50% (32, 46%) of patients who experienced a thromboembolic event was diagnosed ante mortem [16].

In our work, a longer ECMO duration was associated with thrombosis, which was shown earlier [33,36]. Moreover, Fisser et al. and Trudzinski et al. found a correlation between shorter aPTT and thrombosis [33,36], which is in contrast to this study in which patients with and without thrombosis showed comparable values of aPTT.

Our in-hospital mortality of 39% is lower compared to a recent analysis of more than 45,000 ECMO cases in Germany, which demonstrated a mortality as high as 54% [37]. Interestingly, thrombotic events did not seem to influence survival after ECMO support. On the contrary, hemorrhage has been shown to increase the risk for mortality by up to 15%, as demonstrated by data from our group and the available literature [10,21,38,39,40].

### 4.2. Thrombotic Events and Type of Anticoagulation

Recently, comparable rates of thrombotic events have been shown either with UFH or with argatroban, which is in line with our work [30]. However, the patients with argatroban showed a trend towards more thrombotic events as compared to the patients receiving UFH. As these patients were sicker and more often experienced sepsis (with negative HIT 2 diagnostic), the question arises if a more pronounced inflammation may be a possible reason.

The patients without anticoagulation during ECMO support had higher SAPS III scores when compared to the other patients with a very high share of them requiring V-A ECMO support. Moreover, this group experienced major bleedings twice as often as all other patients, and 48% of patients died during their ICU stay, which may be attributable to the severity of the underlying disease and severe coagulopathy.

### 4.3. Thrombotic Events and ECMO Configuration

Clearly, the different underlying etiologies of V-A and V-V ECMO support impede comparability of the thrombosis incidence. However, a recent retrospective work from the US demonstrated four-times higher rates of deep vein thrombosis in V-V ECMO support as compared to V-A ECMO support [41]. From a pathophysiological perspective, patients receiving V-V ECMO support and who have severe respiratory failure may suffer from strong inflammation, making development of thrombosis more likely (thromboinflammation) [17,18,29,42]. In contrast to that, we found comparable rates of thrombosis in both ECMO types, which is in line with the French HECTIC trial [20] and recent German work [30]. Interestingly, we found nearly twice as many thrombotic events in the venous system with V-V ECMO support as compared to V-A ECMO support (70% vs. 38%). A possible reason for this could be the far lower blood velocity in the venous bed and the two venous cannulas used in V-V ECMO support.

### 4.4. The Role of Potentially Modifiable Factors

The majority of risk factors for thrombosis in patients needing ECMO support are not modifiable (obesity, smoking, history of hypertension, etc.). Thus, the anticoagulation regime remains the pivotal modifiable factor [43,44]. Different approaches for the reduction of adverse events are discussed in the literature. Given a higher rate of venous thrombosis in patients receiving V-V ECMO support, strict adherence to an institutional anticoagulation protocol in such patients seems reasonable. This holds especially true in patients with COVID-19. Moreover, patients receiving V-A ECMO support and who have cardiac stunning may be at a greater risk for coagulopathy induced by the underlying critical illness. This could justify lowering anticoagulation targets for the first 48 h until resolution of the cardiac stunning.

Furthermore, an ideal parameter for monitoring anticoagulation is still not available, as the anticoagulation monitoring in critically ill patients can be influenced by different factors [19,45]. The ideal anticoagulant, capable to reduce or even eliminate both the risk of bleeding and thrombosis, still does not exist. The newest evidence from preclinical studies shows that antibodies targeting certain coagulation factors may improve efficacy and safety, but data from human studies are still lacking [46,47,48].

Additional risk factors for thrombosis include comorbidity, surgical interventions, invasive procedures, acute illness-related hypercoagulability and immobilization due to sedation [31,49]. In patients receiving ECMO support, surgical trauma, exposure to artificial surfaces, use of multiple indwelling catheters and ECMO cannulas, as mentioned above, further increase the risk for thrombosis [29]. For this reason, the use of an anticoagulation protocol that works best at the respective centers is recommended by the ELSO [19]. Moreover, in the last decade, an individualized approach has been popularized [50]. Whilst it is challenging to isolate the relative influence of specific patient-related factors for thrombosis during extracorporeal life support, failure to do so could result in a missed opportunity for intervention.

As the ECMO duration was associated with thrombosis development, we recommend adherence to institutional ECMO-weaning protocols. Assessing patients for recovery of cardiac and pulmonary function on a daily basis may aid early decannulation. This may contribute to a reduction in adverse events and, thereby, improve outcomes and reduce costs [51,52].

### 4.5. Limitations

This work has several limitations. Due to the retrospective nature of the study, a selection bias could occur. However, all consecutive patients requiring ECMO support from the three ICUs of our department meeting the inclusion criteria were included in the final analysis. Although it may be difficult to discriminate ECMO-related complications from potential complications of the underlying diseases, the reported thrombotic events are most probably a consequence of the ECMO support and prothrombotic state of the organism. We employed the ELSO bleeding definition to classify bleeding, but specific recommendations for the reporting on thrombotic events are still missing. Therefore, we reported on thrombotic events in more detail while providing additional information in the subgroup analyses and Supplementary Material. The most important limitation of this study is related to its nature, as the retrospective identification of thrombosis needs radiological findings. This may lead to an underestimation of thrombotic events in our work. However, due to a relatively liberal approach towards diagnostic modalities and post-mortem examinations, this chance may be rather small. Moreover, due to the lack of certain clinical manifestations, the day of thrombosis detection may not be the exact day of thrombosis development. As we were not able to retrieve the type of cannulation (central or periphery) in our work, the influence of these factors on the occurrence of adverse events could not be evaluated. Lastly, our study comprises a comparably large cohort of critically ill patients requiring ECMO support, but larger samples and further studies are needed to clarify the risk factors for thrombotic events and possible prevention of thrombosis during ECMO support.

## 5. Conclusions

ECMO is a specialized life-support modality with a high risk of complications. We found longer ECMO duration to be associated with thrombosis occurrence. Following hemorrhage, thromboembolic complications are common adverse events. However, in contrast to major bleeding, no impact on mortality was observed. The question arises if a protocol with less anticoagulation may have a role to play in the future.

## Figures and Tables

**Table 1:** Demographic and clinical characteristics of the included patients (n = 417).

Patient Characteristics	All Patients (n = 417)	No Thrombotic Events (n = 325)	Thrombotic Events (n = 92)	*p* Value
Age (years)	61.0 (11–87)	61.0 (11–85)	63.0 (20–87)	0.264
Sex (male)	298 (71.5)	233 (71.7)	65 (70.7)	0.896
Height (cm)	172 ± 9.8	172 ± 10.1	171 ± 8.9	0.360
Weight (kg)	80.7 ± 17.8	80.7 ± 18.3	80.6 ± 15.4	0.951
Body mass index (kg/m^2^)	27.1 ± 5.2	27.1 ± 5.3	27.4 ± 4.8	0.586
SAPS III score	67 (28–117)	67 (28–117)	68 (40–104)	0.223
SAPS III score predicted mortality (%)	50 (1–96)	50 (1–96)	52 (6–93)	0.223
SOFA score	12 (2–21)	12 (2–21)	12 (3–20)	0.974
SOFA respiratory	2 (0–4)	3 (0–4)	2 (0–4)	0.634
SOFA neurology	4 (0–4)	4 (0–4)	4 (0–4)	0.976
SOFA cardiovascular	4 (0–4)	4 (0–4)	4 (0–4)	0.472
SOFA liver	0 (0–4)	0 (0–4)	1 (0–4)	0.105
SOFA coagulation	1 (0–4)	1 (0–4)	1 (0–3)	0.863
SOFA renal	1 (0–4)	1 (0–4)	1 (0–4)	0.725
CPR before ECMO initiation	86 (20.6)	70 (21.5)	16 (17.4)	0.466
Length of ICU stay (days)	18 (1–170)	17 (1–170)	21 (1–98)	0.018
ICU admission reason				
Cardiac non-surgical	205 (49.2)	153 (47.1)	52 (56.5)	0.514
Respiratory failure	108 (25.9)	85 (26.2)	23 (25.0)	
Cardiac surgery	90 (21.6)	75 (23.1)	15 (16.3)	
Hypothermia	11 (2.6)	9 (2.8)	2 (2.2)	
Trauma	3 (0.7)	3 (0.9)	0 (0.0)	

Values are presented as the mean ± SD, median (minimum–maximum) or number (%) of patients. ECMO: extracorporeal membrane oxygenation; SOFA: sequential organ failure assessment score; SAPS III: simplified acute physiology score III; ICU: intensive care unit; CPR: cardiopulmonary resuscitation.

**Table 2:** ECMO-related characteristics and outcomes according to the occurrence of thrombotic events (n = 417).

Clinical Characteristics	All Patients (n = 417)	No Thrombotic Events (n = 325)	Thrombotic Events (n = 92)	*p* Value
ECMO support indications				
Cardiogenic shock	290 (69.5)	225 (69.2)	65 (70.7)	0.970
Respiratory failure	116 (27.8)	91 (28.0)	25 (27.2)	
Rewarming	11 (2.6)	9 (2.8)	2 (2.2)	
Type of ECMO support				
Veno-arterial	315 (75.5)	246 (75.7)	69 (75.0)	0.891
Veno-venous	102 (24.5)	79 (24.3)	23 (25.0)	
Surgical intervention	227 (54.4)	178 (54.8)	49 (53.3)	0.813
ECMO related clinical course				
ECMO support duration (days)	6 (1–46) 7.5 ± 6.2	6 (1–46) 6.9 ± 5.6	7 (1–36) 9.4 ± 7.6	0.001
ECMO support duration <7 days	277 (66.4)	226 (65.5)	51 (55.4)	0.013
Days from admission to ECMO initiation	0 (0–36)	0 (0–36)	0 (0–17)	0.259
Anticoagulation during ECMO support				
Unfractionated heparin	296 (71.2)	235 (72.5)	61 (66.3)	0.236
Argatroban	70 (16.8)	49 (15.1)	21 (22.8)	
None	50 (12.0)	40 (12.3)	10 (10.9)	
Reason for ECMO support termination				
Improvement (weaned)	295 (70.7)	227 (69.8)	68 (73.9)	0.928
Bridge to other assistance (heart transplant or ventricular assist device)	18 (4.3)	15 (4.6)	3 (3.3)	
Hemorrhage	7 (1.7)	6 (1.8)	1 (1.1)	
Death	97 (23.3)	77 (23.7)	20 (21.7)	

Values are presented as the mean ± SD or number (%) of patients. ECMO: extracorporeal membrane oxygenation.

**Table 3:** Adverse events and mortality during ECMO support (n = 417).

Complications	All Patients (n = 417)	No Thrombotic Events (n = 325)	Thrombotic Events (n = 92)	*p* Value
Thrombotic events	92 (22.1)	-	92 (100.0)	-
Venous thrombosis	58 (13.9)	-	58 (63.0)	-
Arterial thrombosis	45 (10.8)	-	45 (48.9)	-
Combined thrombosis	24 (5.8)	-	24 (26.1)	-
Pulmonary embolism	10 (2.4)	-	10 (10.9)	-
Thromboembolic stroke	4 (1.0)	-	4 (4.4)	-
Day of diagnosis of thrombosis	6 (1–29)	-	6 (1–29)	-
Thrombosis during ECMO only ^a^	57 (13.7)	-	57 (62.0)	-
Hemorrhage	178 (42.7)	143 (44.0)	35 (38.0)	0.272
Major hemorrhage	106 (25.4)	79 (24.3)	27 (29.3)	0.344
Minor hemorrhage	72 (17.3)	64 (19.7)	8 (8.7)	0.012
Sepsis	85 (20.4)	65 (20.0)	20 (21.7)	0.770
Mortality				
Death during ECMO support	97 (23.3)	77 (23.7)	20 (21.7)	0.780
ICU mortality	148 (35.5)	116 (35.7)	32 (34.8)	0.902
In-hospital mortality	162 (38.8)	127 (39.1)	35 (38.0)	0.904
Three-month mortality	166 (39.8)	130 (40.0)	36 (39.1)	0.905
One-year mortality	173 (41.5)	136 (41.8)	37 (40.2)	0.811
Cause of death (in-hospital mortality)				
MODS	50 (31.1)	38 (30.2)	12 (34.3)	0.637
Cardiac cause	62 (38.5)	46 (36.5)	16 (45.7)	
Brain death	27 (16.8)	24 (19.0)	3 (8.6)	
Sepsis	16 (9.9)	13 (10.3)	3 (8.6)	
Respiratory failure	6 (3.7)	5 (4.0)	1 (2.9)	

^a^ Thrombosis during ECMO only includes all diagnosed events from the moment of cannulation until 24 h after termination of ECMO support. Values are presented as the number (%) of patients. ECMO: extracorporeal membrane oxygenation; ICU: intensive care unit; MODS: Multiple organ dysfunction syndrome.

**Table 4:** Univariate analyses: Variables with potential association with thrombosis (n = 417).

Nondependent Variable	B-Coefficient	*p* Value	HR	95% Confidence Interval	
				Lower	Upper
Age (years)	0.010	0.164	1.01	1.00	1.02
SAPS III score	0.008	0.220	1.01	1.00	1.02
ECMO duration (days)	0.041	0.001	1.04	1.02	1.07
ICU length of stay (days)	0.008	0.075	1.01	1.00	1.02
ECMO Indication (reference category: respiratory failure)					
Cardiogenic shock	0.087	0.710	1.09	0.69	1.73
Rewarming	−0.187	0.799	0.83	0.20	3.50
Type of ECMO	−0.018	0.942	0.98	0.61	1.57
Laboratory parameters (median)					
C-reactive protein (mg/dL)	−0.001	0.954	0.99	0.97	1.03
Procalcitonin (ug/L)	−0.002	0.599	1.00	0.99	1.00
Leukocytes (g/L)	0.031	0.057	1.03	0.99	1.06
Red blood cells (T/L)	0.752	<0.001	2.12	1.51	2.98
Hemoglobin (g/dL)	0.032	<0.001	1.03	1.02	1.10
Platelets (g/L)	0.005	<0.001	1.01	1.00	1.01
International normalized ratio	0.058	0.641	1.06	0.83	1.35
Activated partial thromboplastin time (s)	0.002	0.598	1.00	0.99	1.01
Fibrinogen (mg/dL)	0.000	0.660	1.00	1.00	1.00
Antithrombin (%)	0.002	0.771	1.00	0.99	1.01
InTEM Clotting Time (mm)	0.000	0.954	1.00	0.99	1.00
InTEM Maximal Clot Firmness (mm)	0.033	0.034	1.03	1.00	1.07
FibTEM Maximal Clot Firmness (mm)	0.029	0.032	1.03	1.00	1.06

SAPS III: simplified acute physiology score III; ICU: intensive care unit; ECMO: extracorporeal membrane oxygenation; HR: hazard ratios.

**Table 5:** Multivariate analysis: Identification of risk factors for thrombotic events (n = 417).

Variable	B-Coefficient	*p* Value	HR	95% Confidence Interval	
				Lower	Upper
ECMO duration (days)	0.040	0.037	1.04	1.00	1.08
Red blood cells (T/L)	0.602	0.170	1.83	0.77	4.31
Leukocytes (g/L)	0.006	0.807	1.01	0.96	1.06
Platelets (g/L)	0.004	0.103	1.00	0.99	1.01
FibTEM Maximal Clot Firmness (mm)	0.018	0.296	1.02	0.99	1.05

ECMO: extracorporeal membrane oxygenation; HR: hazard ratio.

## Data Availability

The datasets used and analyzed during the current study are made available from the corresponding author on reasonable request.

## Supplementary Materials

The following supporting information can be downloaded at: https://www.mdpi.com/article/10.3390/jcm12031082/s1, Figure S1. Kaplan–Meier curve: time from ECMO initiation to thrombotic event (n = 417) was 24.8 days for veno-arterial ECMO (n = 315; 95% CI 23.7–25.9) and 25.8 days for veno-venous ECMO (n = 102; 95% CI 24.2–27.5). Maximum observation period for thrombosis occurrence was 30 days from ECMO support initiation. Abbreviations: ECMO: Extracorporeal membrane oxygenation; Table S1. STROBE Statement—Checklist of items that should be included in reports of cohort studies; Table S2. Laboratory parameters during ECMO support in regard to thrombotic event presence (n = 417); Table S3. Univariate analyses: Identification of risk factors and predictors for thrombosis (n = 417); Table S4. Comparison of different anticoagulation approaches (n = 417); Table S5. Patients with thrombotic events: Demographic and clinical characteristics in regard to ECMO type (n = 92).

## References

[1] Combes A., Hajage D., Capellier G., Demoule A., Lavoué S., Guervilly C., Da Silva D., Zafrani L., Tirot P., Veber B. (2018). Extracorporeal Membrane Oxygenation for Severe Acute Respiratory Distress Syndrome. N. Engl. J. Med..

[2] Combes A., Pesenti A., Ranieri V.M. (2017). Fifty Years of Research in ARDS. Is Extracorporeal Circulation the Future of Acute Respiratory Distress Syndrome Management?. Am. J. Respir. Crit. Care Med..

[3] Davies A., Jones D., Bailey M., Beca J., Bellomo R., Blackwell N., Forrest P., Gattas D., Granger E., Herkes R. (2009). Extracorporeal Membrane Oxygenation for 2009 Influenza A(H1N1) Acute Respiratory Distress Syndrome. JAMA.

[4] El Sibai R., Bachir R., El Sayed M. (2018). Outcomes in Cardiogenic Shock Patients with Extracorporeal Membrane Oxygenation Use: A Matched Cohort Study in Hospitals across the United States. BioMed Res. Int..

[5] Hajjar L.A., Teboul J.-L. (2019). Mechanical Circulatory Support Devices for Cardiogenic Shock: State of the Art. Crit. Care.

[6] Peek G.J., Mugford M., Tiruvoipati R., Wilson A., Allen E., Thalanany M.M., Hibbert C.L., Truesdale A., Clemens F., Cooper N. (2009). Efficacy and economic assessment of conventional ventilatory support versus extracorporeal membrane oxygenation for severe adult respiratory failure (CESAR): A multicentre randomised controlled trial. Lancet.

[7] Pham T., Combes A., Rozé H., Chevret S., Mercat A., Roch A., Mourvillier B., Ara-Somohano C., Bastien O., Zogheib E. (2013). Extracorporeal membrane oxygenation for pandemic influenza A(H1N1)-induced acute respiratory distress syndrome: A cohort study and propensity-matched analysis. Am. J. Respir. Crit. Care Med..

[8] Schmidt M., Zogheib E., Rozé H., Repesse X., Lebreton G., Luyt C.E., Trouillet J.L., Bréchot N., Nieszkowska A., Dupont H. (2013). The PRESERVE mortality risk score and analysis of long-term outcomes after extracorporeal membrane oxygenation for severe acute respiratory distress syndrome. Intensive Care Med..

[9] Thompson B.T., Chambers R.C., Liu K.D. (2017). Acute Respiratory Distress Syndrome. N. Engl. J. Med..

[10] Treml B., Breitkopf R., Bukumirić Z., Bachler M., Boesch J., Rajsic S. (2022). ECMO Predictors of Mortality: A 10-Year Referral Centre Experience. J. Clin. Med..

[11] Zavalichi M.A., Nistor I., Nedelcu A.E., Zavalichi S.D., Georgescu C.M.A., Stătescu C., Covic A. (2020). Extracorporeal Membrane Oxygenation in Cardiogenic Shock due to Acute Myocardial Infarction: A Systematic Review. BioMed Res. Int..

[12] Zangrillo A., Landoni G., Biondi-Zoccai G., Greco M., Greco T., Frati G., Patroniti N., Antonelli M., Pesenti A., Pappalardo F. (2013). A meta-analysis of complications and mortality of extracorporeal membrane oxygenation. Crit. Care Resusc. J. Australas. Acad. Crit. Care Med..

[13] Arachchillage D.J., Rajakaruna I., Scott I., Gaspar M., Odho Z., Banya W., Vlachou A., Isgro G., Cagova L., Wade J. (2021). Impact of major bleeding and thrombosis on 180-day survival in patients with severe COVID-19 supported with veno-venous extracorporeal membrane oxygenation in the United Kingdom: A multicentre observational study. Br. J. Haematol..

[14] Cheng A., Sun H.Y., Tsai M.S., Ko W.J., Tsai P.R., Hu F.C., Chen Y.C., Chang S.C. (2016). Predictors of survival in adults undergoing extracorporeal membrane oxygenation with severe infections. J. Thorac. Cardiovasc. Surg..

[15] Parzy G., Daviet F., Puech B., Sylvestre A., Guervilly C., Porto A., Hraiech S., Chaumoitre K., Papazian L., Forel J.M. (2020). Venous Thromboembolism Events Following Venovenous Extracorporeal Membrane Oxygenation for Severe Acute Respiratory Syndrome Coronavirus 2 Based on CT Scans. Crit. Care Med..

[16] Rastan A.J., Lachmann N., Walther T., Doll N., Gradistanac T., Gommert J.F., Lehmann S., Wittekind C., Mohr F.W. (2006). Autopsy findings in patients on postcardiotomy extracorporeal membrane oxygenation (ECMO). Int. J. Artif. Organs.

[17] Lim M.S., McRae S. (2021). COVID-19 and immunothrombosis: Pathophysiology and therapeutic implications. Crit. Rev. Oncol. /Hematol..

[18] Ranucci M., Ballotta A., Di Dedda U., Baryshnikova E., Dei Poli M., Resta M., Falco M., Albano G., Menicanti L. (2020). The procoagulant pattern of patients with COVID-19 acute respiratory distress syndrome. J. Thromb. Haemost. JTH.

[19] Laurance Lequier G.A., Al-Ibrahim O., Bembea M., Brodie D., Brogan T., Buckvold S., Chicoine L., Conrad S., Cooper D., Dalton H. (2014). ELSO Anticoagulation Guideline.

[20] Cartwright B., Bruce H.M., Kershaw G., Cai N., Othman J., Gattas D., Robson J.L., Hayes S., Alicajic H., Hines A. (2021). Hemostasis, coagulation and thrombin in venoarterial and venovenous extracorporeal membrane oxygenation: The HECTIC study. Sci. Rep..

[21] Rajsic S., Breitkopf R., Oezpeker U.C., Bukumirić Z., Dobesberger M., Treml B. (2022). The Role of Excessive Anticoagulation and Missing Hyperinflammation in ECMO-Associated Bleeding. J. Clin. Med..

[22] Aubron C., DePuydt J., Belon F., Bailey M., Schmidt M., Sheldrake J., Murphy D., Scheinkestel C., Cooper D.J., Capellier G. (2016). Predictive factors of bleeding events in adults undergoing extracorporeal membrane oxygenation. Ann. Intensive Care.

[23] Weir-McCall J.R., Galea G., Mun Mak S., Joshi K., Agrawal B., Screaton N., Toshner M., Ruggiero A., Benedetti G., Brozik J. (2021). Vascular Thrombosis in Severe Coronavirus Disease 2019 Requiring Extracorporeal Membrane Oxygenation: A Multicenter Study. Crit. Care Med..

[24] Ripoll B., Rubino A., Besser M., Patvardhan C., Thomas W., Sheares K., Shanahan H., Agrawal B., Webb S., Vuylsteke A. (2021). Observational study of thrombosis and bleeding in COVID-19 VV ECMO patients. Int. J. Artif. Organs.

[25] Yusuff H., Zochios V., Brodie D. (2020). Thrombosis and Coagulopathy in COVID-19 Patients Requiring Extracorporeal Membrane Oxygenation. ASAIO J..

[26] Capodanno D., Angiolillo D.J. (2013). Management of antiplatelet therapy in patients with coronary artery disease requiring cardiac and noncardiac surgery. Circulation.

[27] Schlimp C., Schöchl H., Alber H.J. (2018). Empfehlung der Arbeitsgruppe Perioperative Gerinnung der ÖGARI zum Thema: Perioperatives Management von PatientInnen mit Koronarstents unter Dualer Plättchenhemmung bei Nicht-Kardiochirurgischen Eingriffen.

[28] Kaplan D., Casper T.C., Elliott C.G., Men S., Pendleton R.C., Kraiss L.W., Weyrich A.S., Grissom C.K., Zimmerman G.A., Rondina M.T. (2015). VTE Incidence and Risk Factors in Patients With Severe Sepsis and Septic Shock. Chest.

[29] Millar J.E., Fanning J.P., McDonald C.I., McAuley D.F., Fraser J.F. (2016). The inflammatory response to extracorporeal membrane oxygenation (ECMO): A review of the pathophysiology. Crit. Care.

[30] Fisser C., Winkler M., Malfertheiner M.V., Philipp A., Foltan M., Lunz D., Zeman F., Maier L.S., Lubnow M., Müller T. (2021). Argatroban versus heparin in patients without heparin-induced thrombocytopenia during venovenous extracorporeal membrane oxygenation: A propensity-score matched study. Crit. Care.

[31] Tran A., Fernando S.M., Rochwerg B., Cook D.J., Crowther M.A., Fowler R.A., Alhazzani W., Siegal D.M., Castellucci L.A., Zarychanski R. (2022). Prognostic Factors Associated With Development of Venous Thromboembolism in Critically Ill Patients-A Systematic Review and Meta-Analysis. Crit. Care Med..

[32] Stokes J.W., Gannon W.D., Sherrill W.H., Armistead L.B., Bacchetta M., Rice T.W., Semler M.W., Casey J.D. (2020). Bleeding, Thromboembolism, and Clinical Outcomes in Venovenous Extracorporeal Membrane Oxygenation. Crit. Care Explor..

[33] Fisser C., Reichenbächer C., Müller T., Schneckenpointner R., Malfertheiner M.V., Philipp A., Foltan M., Lunz D., Zeman F., Lubnow M. (2019). Incidence and Risk Factors for Cannula-Related Venous Thrombosis After Venovenous Extracorporeal Membrane Oxygenation in Adult Patients With Acute Respiratory Failure. Crit. Care Med..

[34] Abruzzo A., Gorantla V., Thomas S.E. (2022). Venous thromboembolic events in the setting of extracorporeal membrane oxygenation support in adults: A systematic review. Thromb. Res..

[35] Mazzeffi M., Greenwood J., Tanaka K., Menaker J., Rector R., Herr D., Kon Z., Lee J., Griffith B., Rajagopal K. (2016). Bleeding, Transfusion, and Mortality on Extracorporeal Life Support: ECLS Working Group on Thrombosis and Hemostasis. Ann. Thorac. Surg..

[36] Trudzinski F.C., Minko P., Rapp D., Fähndrich S., Haake H., Haab M., Bohle R.M., Flaig M., Kaestner F., Bals R. (2016). Runtime and aPTT predict venous thrombosis and thromboembolism in patients on extracorporeal membrane oxygenation: A retrospective analysis. Ann. Intensive Care.

[37] Friedrichson B., Mutlak H., Zacharowski K., Piekarski F. (2021). Insight into ECMO, mortality and ARDS: A nationwide analysis of 45,647 ECMO runs. Crit. Care.

[38] Rajsic S., Treml B., Jadzic D., Breitkopf R., Oberleitner C., Popovic Krneta M., Bukumiric Z. (2022). Extracorporeal membrane oxygenation for cardiogenic shock: A meta-analysis of mortality and complications. Ann. Intensive Care.

[39] Baldetti L., Nardelli P., Ajello S., Melisurgo G., Calabrò M.G., Pieri M., Scandroglio A.M. (2022). Anti-thrombotic Therapy with Cangrelor and Bivalirudin in Venoarterial Extracorporeal Membrane Oxygenation Patients Undergoing Percutaneous Coronary Intervention: A Single-Center Experience. ASAIO J. (Am. Soc. Artif. Intern. Organs 1992).

[40] Katz A., Lewis T.C., Arnouk S., Altshuler D., Papadopoulos J., Toy B., Smith D.E., Merchan C. (2021). Clinical Use of Cangrelor After Percutaneous Coronary Intervention in Patients Requiring Mechanical Circulatory Support. Ann. Pharmacother..

[41] Vazquez Z.G.S., Sodha N.R., Devers C., Ventetuolo C.E., Abbasi A. (2021). Prevalence of Deep Vein Thrombosis in Patients Supported With Extracorporeal Membrane Oxygenation. ASAIO J..

[42] Al-Fares A., Pettenuzzo T., Del Sorbo L. (2019). Extracorporeal life support and systemic inflammation. Intensive Care Med. Exp..

[43] Goldhaber S.Z. (2010). Risk Factors for Venous Thromboembolism. J. Am. Coll. Cardiol..

[44] Cushman M. (2007). Epidemiology and risk factors for venous thrombosis. Semin. Hematol..

[45] Rajsic S., Breitkopf R., Bachler M., Treml B. (2021). Diagnostic Modalities in Critical Care: Point-of-Care Approach. Diagnostics.

[46] DeLoughery E.P., Olson S.R., Puy C., McCarty O.J.T., Shatzel J.J. (2019). The Safety and Efficacy of Novel Agents Targeting Factors XI and XII in Early Phase Human Trials. Semin. Thromb. Hemost..

[47] Wallisch M., Lorentz C.U., Lakshmanan H.H.S., Johnson J., Carris M.R., Puy C., Gailani D., Hinds M.T., McCarty O.J.T., Gruber A. (2020). Antibody inhibition of contact factor XII reduces platelet deposition in a model of extracorporeal membrane oxygenator perfusion in nonhuman primates. Res. Pract. Thromb. Haemost..

[48] Rajsic S., Breitkopf R., Jadzic D., Popovic Krneta M., Tauber H., Treml B. (2022). Anticoagulation Strategies during Extracorporeal Membrane Oxygenation: A Narrative Review. J. Clin. Med..

[49] Eck R.J., Hulshof L., Wiersema R., Thio C.H.L., Hiemstra B., van den Oever N.C.G., Gans R.O.B., van der Horst I.C.C., Meijer K., Keus F. (2021). Incidence, prognostic factors, and outcomes of venous thromboembolism in critically ill patients: Data from two prospective cohort studies. Crit. Care.

[50] Welsby I., Ortel T.L. (2015). Is it time for individualized thromboprophylaxis regimens in the ICU?. Crit. Care Med..

[51] Gannon W.D., Stokes J.W., Bloom S., Sherrill W., Bacchetta M., Rice T.W., Semler M.W., Casey J.D. (2021). Safety and Feasibility of a Protocolized Daily Assessment of Readiness for Liberation From Venovenous Extracorporeal Membrane Oxygenation. Chest.

[52] Pratt E.H., Mausert S., Wilson M.D., Emerson L.J., Navuluri N., Pulsipher A.M., Brucker A., Green C.L., Bonadonna D.K., Bryner B.S. (2021). A Daily, Respiratory Therapist Assessment of Readiness to Liberate From Venovenous Extracorporeal Membrane Oxygenation in Patients With Acute Respiratory Distress Syndrome. Crit. Care Explor..

