# Supplementary material for: Thrombotic Events Develop in 1 Out of 5 Patients Receiving ECMO Support: An 11-Year Referral Centre Experience
Source: J Clin Med. 2023 Jan 30;12(3):1082. doi: 10.3390/jcm12031082 (PMC9917555; doi:10.3390/jcm12031082)
Supplement: Supplementary file 1 [file jcm-12-01082-s001.zip › jcm-2120081-supplementary.pdf]

Supplementary material

Thrombotic Events Develop in 1 Out of 5 Patients Receiving ECMO Support: An 11-Year Referral Centre Experience

Sasa Rajsic, Robert Breitkopf, Christopher Rugg, Zoran Bukumirić, Jakob Reitbauer and Benedikt Treml

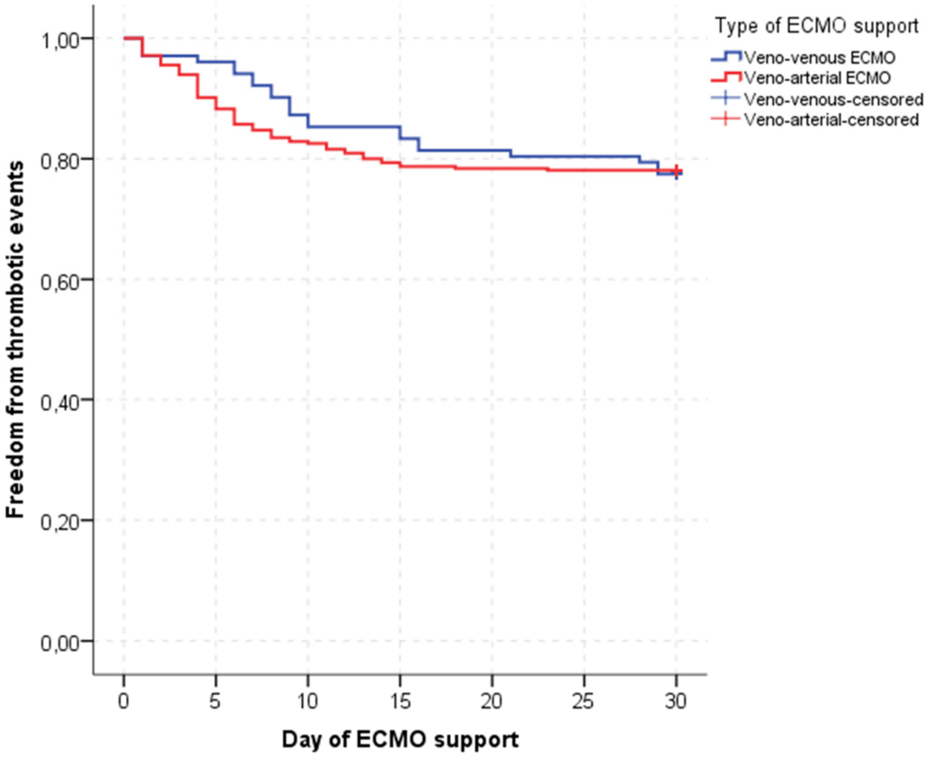

Figure S1. Kaplan-Meier curve: time from ECMO initiation to thrombotic event (n = 417) was 24.8 days for veno-arterial ECMO (n = 315; 95% CI 23.7 - 25.9) and 25.8 days for veno-venous ECMO (n = 102; 95% CI 24.2 - 27.5). Maximum observation period for thrombosis occurrence was 30 days from ECMO support initiation. Abbreviations: ECMO: Extracorporeal membrane oxygenation

Table S1. STROBE Statement - Checklist of items that should be included in reports of cohort studies

No.	Item	Recommendation	Page
	**Title and abstract**		
1		(*a*) Indicate the study's design with a commonly used term in the title or the abstract	1
		(*b*) Provide in the abstract an informative and balanced summary of what was done and what was found	2
	**Introduction**		
2	Background/rationale	Explain the scientific background and rationale for the investigation being reported	3
3	Objectives	State specific objectives, including any prespecified hypotheses	3
	**Methods**		
4	Study design	Present key elements of study design early in the paper	4
5	Setting	Describe the setting, locations, and relevant dates, including periods of recruitment, exposure, follow-up, and data collection	4-5
6	Participants	(*a*) Give the eligibility criteria, and the sources and methods of selection of participants. Describe methods of follow-up	4-5
		(*b*) For matched studies, give matching criteria and number of exposed and unexposed	
7	Variables	Clearly define all outcomes, exposures, predictors, potential confounders, and effect modifiers. Give diagnostic criteria, if applicable	4-5
8*	Data sources/ measurement	For each variable of interest, give sources of data and details of methods of assessment (measurement). Describe comparability of assessment methods if there is more than one group	4-5
9	Bias	Describe any efforts to address potential sources of bias	4-5
10	Study size	Explain how the study size was arrived at	NA
11	Quantitative variables	Explain how quantitative variables were handled in the analyses. If applicable, describe which groupings were chosen and why	4-5
12	Statistical methods	(*a*) Describe all statistical methods, including those used to control for confounding	5
		(*b*) Describe any methods used to examine subgroups and interactions	5
		(*c*) Explain how missing data were addressed	5
		(*d*) If applicable, explain how loss to follow-up was addressed	NA
		(*e*) Describe any sensitivity analyses	NA
	**Results**		
13*	Participants	(a) Report numbers of individuals at each stage of study—eg numbers potentially eligible, examined for eligibility, confirmed eligible, included in the study, completing follow-up, and analysed	6-7
		(b) Give reasons for non-participation at each stage	Fig. 1
		(c) Consider use of a flow diagram	Fig. 1
14*	Descriptive data	(a) Give characteristics of study participants (eg demographic, clinical, social) and information on exposures and potential confounders	Table 1
		(b) Indicate number of participants with missing data for each variable of interest	Tables
		(c) Summarise follow-up time (eg, average and total amount)	Tables
15*	Outcome data	Report numbers of outcome events or summary measures over time	Tables
16	Main results	(*a*) Give unadjusted estimates and, if applicable, confounder-adjusted estimates and their precision (eg, 95% confidence interval). Make clear which confounders were adjusted for and why they were included	Tables
		(*b*) Report category boundaries when continuous variables were categorized	Tables
		(*c*) If relevant, consider translating estimates of relative risk into absolute risk for a meaningful time period	NA
17	Other analyses	Report other analyses done—eg analyses of subgroups and interactions, and sensitivity analyses	6-7
	**Discussion**		
18	Key results	Summarise key results with reference to study objectives	8
19	Limitations	Discuss limitations of the study, taking into account sources of potential bias or imprecision. Discuss both direction and magnitude of any potential bias	10

20	Interpretation	Give a cautious overall interpretation of results considering objectives, limitations, multiplicity of analyses, results from similar studies, and other relevant evidence	8-10
21	Generalisability	Discuss the generalisability (external validity) of the study results	8-10
	**Other information**		
22	Funding	Give the source of funding and the role of the funders for the present study and, if applicable, for the original study on which the present article is based	Title page

*Give information separately for exposed and unexposed groups.

Table S2. Laboratory parameters during ECMO support in regard of thrombotic event presence (n = 417)

Laboratory parameters	All patients (n = 417)	No thrombotic events (n = 325)	Thrombotic events (n = 92)	P value
Hemoglobin (g/dL)	9.3 ± 1.2	9.2 ± 1.0	9.9 ± 1.5	0.001
Red blood cells (T/L)	3.1 ± 0.4	3.1 ± 0.4	3.3 ± 0.5	0.022
Hematocrit (%)	0.28 ± 0.1	0.27 ± 0.1	0.29 ± 0.1	<0.001
Leucocytes (g/L)	10.4 (0.3-46.3)	10.3 (0.3-46.3)	11.2 (1.3-30.1)	0.048
C-reactive protein (mg/L)	9.2 (0.1-42.2)	9.1 (0.1-42.2)	9.5 (0.1-35.3)	0.861
Procalcitonin (ug/L)	3.4 (0.1-1137.0)	3.4 (0.1-1137.0)	4.0 (0.1-187.3)	0.382
Platelets (g/L)	79.0 (12-378)	76.0 (12-378)	95.5 (27-364)	0.005
International normalized ratio	1.4 (0.8-6)	1.4 (0.8-6)	1.4 (1.1-6)	0.691
Activated partial thromboplastin time (s)	54.0 (27-201)	54.0 (27-201)	53.0 (29-201)	0.289
Prothrombin time (%)	58.0 (9-104)	57.5 (9-104)	58.5 (9-97.5)	0.822
Fibrinogen (mg/dL)	323.0 (39-918)	327.0 (39-918)	310.1 (39-813)	0.783
Antithrombin (%)	52.0 (19-121)	52.0 (19-121)	52.0 (19-121)	0.792
Factor XIII (%)	56.8 (16-130)	56.8 (16-129)	56.0 (17.5-130)	0.580
InTEM Clotting Time (s)	215.0 (56.5-753)	214.5 (56.5-753)	217.5 (138-465.5)	0.624
InTEM Maximal Clot Firmness (mm)	51.5 (5-82)	51.0 (5-73)	53.0 (33-82)	0.112
FibTEM Maximal Clot Firmness (mm)	15.3 (2-59)	15.0 (2-59)	18.0 (6-58)	0.085

Values are presented as mean ± SD or median (minimum - maximum). Laboratory parameters are presented as median over the whole ECMO period for patient without thrombotic event, and as median over the time before thrombotic event.

Table S3. Univariate analyses: Identification of risk factors and predictors for thrombosis (Continuation of Table 4, n = 417)

Nondependent variable	B-coefficient	P value	HR	95% confidence interval lower	upper
Sex (male/female)	0.024	0.915	1.03	0.66	1.61
Observation period (year)	-0.036	0.283	0.96	0.90	1.03
SOFA Score	0.003	0.922	1.00	0.95	1.06
CPR before ECMO	-0.218	0.428	0.81	0.47	1.38
Admission reason (reference category: cardiac non-surgical)					
Cardiac surgery	-0.487	0.101	0.62	0.35	1.09
Respiratory disease	-0.241	0.336	0.76	0.48	1.28
Trauma	-11.198	0.969	0.00	-	-
Hypothermia	-0.421	0.559	0.66	0.16	2.70
ECMO Indication (reference category: respiratory failure)					
Cardiogenic shock	0.087	0.710	1.09	0.69	1.73
Rewarming	-0.187	0.799	0.83	0.20	3.50
Type of ECMO	-0.018	0.942	0.98	0.61	1.57
Complications					
Hemorrhage	-0.220	0.305	0.80	0.53	1.22
Sepsis	0.038	0.881	1.04	0.63	1.71
Anticoagulation regime					
Anticoagulation medication (yes)	0.097	0.771	1.10	0.57	2.12
Anticoagulation with Argatroban	0.409	0.101	1.50	0.92	2.44
Anticoagulation with UFH	-0.254	0.249	0.78	0.51	1.20
No anticoagulation	-0.097	0.771	0.91	0.47	1.75
Laboratory parameters (median)					
C-reactive protein (mg/dL)	-0.001	0.954	0.99	0.97	1.03
Procalcitonin (ug/L)	-0.002	0.599	1.00	0.99	1.00
Leucocites (g/L)	0.031	0.057	1.03	0.99	1.06
Red blood cells (T/L)	0.752	<0.001	2.12	1.51	2.98
Hemoglobin (g/dL)	0.032	<0.001	1.03	1.02	1.10
Platelets (g/L)	0.005	<0.001	1.01	1.00	1.01
International normalized ratio	0.058	0.641	1.06	0.83	1.35
Activated partial thromboplastin time (s)	0.002	0.598	1.00	0.99	1.01
Fibrinogen (mg/dL)	0.000	0.660	1.00	1.00	1.00
Antithrombin (%)	0.002	0.771	1.00	0.99	1.01
InTEM Clotting Time (mm)	0.000	0.954	1.00	0.99	1.00
InTEM Maximal Clot Firmness (mm)	0.033	0.034	1.03	1.00	1.07
FibTEM Maximal Clot Firmness (mm)	0.029	0.032	1.03	1.00	1.06

SOFA: sequential organ failure assessment score; ECMO: extracorporeal membrane oxygenation; UFH: Unfractionated Heparin; CPR: cardiopulmonary resuscitation

Table S4. Comparison of different anticoagulation approaches (n = 417)

Characteristics	UFH (n = 296)	Argatroban (n = 70)	None (n = 50)	P value[1]	UFH vs Ar	UFH vs None	Ar vs None
Age (years)	61.5 (11-85)	58.0 (20-79)	65.0 (14-87)	0.246			
SAPS III score	66 (30-117)	70.5 (28-112)	72 (42-103)	0.016	0.072	0.109	1.000
SOFA score	12 (2-20)	12 (4-19)	12 (5-21)	0.256			
Length of ICU stay (days)	19 (1-170)	21 (1-98)	6.5 (1-81)	<0.001	0.410	<0.001	<0.001
CPR before ECMO initiation	56 (18.9)	15 (21.4)	15 (30.0)	0.199			
ECMO support duration (days)	6 (1-36)	7.5 (1-46)	2.5 (1-19)	<0.001	0.020	<0.001	<0.001
Veno-arterial ECMO	227 (76.7)	43 (61.4)	45 (90.0)	0.001	0.015	0.039	<0.001
Veno-venous ECMO	69 (23.3)	27 (38.6)	5 (10.0)				
ECMO support indications							
Cardiogenic shock	212 (71.6)	41(58.6)	37 (74.0)	0.051			
Respiratory failure	77 (26.0)	28 (40.0)	10 (20.0)				
Rewarming	7 (2.4)	1 (1.4)	3 (6.0)				
Complications							
Thrombosis	61 (20.6)	21 (30.0)	10 (20.0)	0.218			
Thrombosis arterial	31 (10.5)	7 (10.0)	7 (14.0)	0.737			
Thrombosis venous	36 (12.2)	15 (21.4)	7 (14.0)	0.132			
Thrombosis combined	14 (4.7)	6 (8.6)	4 (8.0)	0.304			
Hemorrhage	122 (41.2)	27 (38.6)	29 (58.0)	0.063			
Major hemorrhage	70 (23.6)	15 (21.4)	21 (42.0)	0.016	0.755	0.009	0.026
Minor hemorrhage	52 (17.6)	12 (17.1)	8 (16.0)	0.963			
Sepsis	54 (18.2)	23 (32.9)	7 (14.0)	0.012	0.009	0.552	0.020
Mortality							
Death during ECMO	59 (19.9)	13 (18.6)	24 (48.0)	<0.001	0.869	<0.001	0.001
ICU mortality	94 (31.8)	24 (34.3)	29 (58.0)	0.002	0.673	<0.001	0.015
One-year mortality	111 (37.5)	28 (40.0)	33 (66.0)	0.001	0.784	<0.001	0.006

Values are presented as median (minimum - maximum) or number (%) of patients. [1]p-value for UFH, Argatroban and none comparison; UFH: Unfractionated Heparin; Ar: Argatroban; SAPS III: simplified acute physiology score III; SOFA: sequential organ failure assessment score; ICU: intensive care unit; ECMO: extracorporeal membrane oxygenation; CPR: cardiopulmonary resuscitation: MODS: multiple organ dysfunction syndrome.

Supplementary Table S5. Patients with thrombotic events: Demographic and clinical characteristics in regard of ECMO type (n = 92)

Patient characteristics	va-ECMO (n = 69)	vv-ECMO (n = 23)	P value
Age (years)	64 (20-87)	51 (26-73)	<0.001
Male sex	48 (69.6)	17 (73.9)	0.795
Body mass index (kg/m²)	26.9 ± 4.5	28.7 ± 5.7	0.191
SAPS III score	69.0 (40-104)	66.5 (48-97)	0.592
SOFA score	12 (3-20)	12 (8-18)	0.572
CPR before ECMO initiation	14 (20.3)	2 (8.7)	0.341
Surgical intervention	44 (63.8)	5 (21.7)	0.001
Length of ICU stay (days)	16 (1-98)	27 (10-92)	0.034
ICU admission reason			
Respiratory failure	3 (4.3)	20 (87.0)	<0.001
Cardiac non-surgical	50 (72.5)	2 (8.7)	
Cardiac surgery	15 (21.7)	0 (0.0)	
Hypothermia	1 (1.4)	1 (4.3)	
ECMO support duration (days)	6 (1-17)	15 (5-36)	<0.001
ECMO support duration <7 days	46 (66.7)	5 (21.7)	<0.001
Days from admission to ECMO initiation	0 (0-2)	0 (0-17)	0.005
Baseline laboratory parameters			
C-reactive protein (mg/dL)	1.3 (0.1-20.1)	13.3 (0.2-40.7)	<0.001
Procalcitonin (ug/L)	0.4 (0.1-46)	1.0 (0.1 - 126)	0.270
Leucocytes (g/L)	10.2 (5.5-31.3)	11.5 (3.0-31.7)	0.942
Fibrinogen (mg/dL)	326 (69-723)	525 (187-1104)	0.031
Thrombotic event localization			
Central venous thrombosis	16 (23.2)	14 (60.9)	0.037
Multiple venous thromboses	3 (4.3)	1 (4.3)	
Peripheral venous thrombosis	7 (10.1)	1 (4.3)	
Central arterial thrombosis	20 (29.0)	3 (13.0)	
Peripheral arterial thrombosis	6 (8.7)	0 (0.0)	
Combined thrombosis	11 (15.9)	4 (17.4)	
ECMO cannula or central catheters	6 (8.7)	0 (0.0)	
Stroke	3 (4.3)	1 (4.3)	1.000
Pulmonary embolism	5 (7.2)	5 (21.7)	0.114
Day of thrombotic event	5 (1-23)	9 (1-29)	0.003
Hemorrhage	27 (39.1)	8 (34.8)	0.807
Major hemorrhage	20 (29.0)	7 (30.4)	1.000
Minor hemorrhage	7 (10.1)	1 (4.3)	0.674
Sepsis	9 (13.0)	11 (47.8)	0.001
Anticoagulation during ECMO support			
Unfractionated heparin	49 (71.0)	12 (52.2)	0.025
Argatroban	11 (15.9)	10 (43.5)	
None	9 (13.0)	1 (4.3)	
Mortality			
ICU mortality	25 (36.2)	7 (30.4)	0.801
One-year mortality	28 (40.6)	9 (39.1)	1.000
Cause of death (in-hospital mortality)			
Multiple organ failure	8 (30.8)	4 (44.4)	0.031
Cardiac cause	15 (57.5)	1 (11.1)	

Patient characteristics	va-ECMO (n = 69)	vv-ECMO (n = 23)	P value
Brain death	1 (3.8)	2 (22.2)	
Sepsis	1 (3.8)	2 (22.2)	
Respiratory failure	1 (3.8)	0 (0.0)	

Values are presented as mean ± SD, median (minimum - maximum), or number (%) of patients. SAPS III: simplified acute physiology score III; SOFA: sequential organ failure assessment score; ICU: intensive care unit; ECMO: extracorporeal membrane oxygenation; CPR: cardiopulmonary resuscitation.
